# Supplementary material for: Approaches used to prevent and reduce the use of restrictive practices on adults with learning disabilities: Protocol for a realist review
Source: PLoS One. 2022 Sep 28;17(9):e0270028. doi: 10.1371/journal.pone.0270028 (PMC9518871; doi:10.1371/journal.pone.0270028)
Supplement: S1 File — (PDF) [file pone.0270028.s002.pdf]

Manchester Metropolitan University

Project LEARN

A realist review of interventions used to prevent and reduce the use of restrictive practices on adults with learning disabilities in NHS and independent sector settings (LEARN)

Protocol v1.0, 13 Oct 2020

Protocol authorised by:

Full name in print<br>Role	Date	Signature
Joy Duxbury, Principle Investigator	13.10.2020	

Project Team

Principal Investigator	Professor Joy Duxbury, Professor of Mental Health, Faculty of Health, Psychology and Social Care, Nursing
Co-investigators	Dr Alina Haines-Delmont, study manager, Nursing Department, Faculty of Health, Psychology and Social Care<br><br>Dr Peter Baker, topic expert, Faculty of Social Sciences, Social Policy, Sociology and Social Research

	Professor John Baker, topic expert, Faculty of Medicine and Health, School of Healthcare<br><br>Dr Melanie Chapman, topic expert and PPI facilitator, Faculty of Health, Psychology and Social Care, Social Care & Social Work<br><br>Mrs Beth Morrison, PPI facilitator and expert by experience, Founder at Positive & Active Behaviour Support<br><br>Mr James Ridley, topic expert and PPI facilitator, Faculty of Health, Social Care & Medicine, Medical School<br><br>Dr Kirstine Szifris, methodology expert, Policy Evaluation & Research Unit, Department of Sociology<br><br>Mrs Michaela Thomson, research practitioner and PPI facilitator, Research and Development Team<br><br>Mr Gary Bourlet, PPI facilitator and expert by experience, Learning Disability England
Study Team	Mr Anthony Tsang, project RA, Nursing Department, Faculty of Health, Psychology and Social Care<br><br>Miss Elaine Craig, project support, Nursing Department, Faculty of Health, Psychology and Social Care

For general queries, supply of study documentation, and collection of data, please contact:
Name: Anthony Tsang
Project role: Main reviewer, contributing to all components of the project
Address: Brooks Building, Birley Fields Campus, 53 Bonsall Street. Manchester, M15 6GX
Tel: 07717141742
E-mail: A.tsang@mmu.ac.uk
Problems relating to this study should be referred, in the first instance, to the Principal Investigator.

Funder
National Institute for Health Research 129524

Contents

1 BACKGROUND 4
1.1 Challenging behaviour and restrictive interventions 4
1.2 Evidence explaining why this research is needed now 4
1 RESEARCH QUESTION(S) AND OBJECTIVES 5
2 RESEARCH DESIGN 6
2.1 Review approach 6
2.2 Theoretical framework 7
2.3 Review strategy 7
2.3.1 Phase I: Formulating mid-level theories in the form of CMO configurations 7
2.3.2 Phase II: Conducting a systematic search of literature 9
2.3.3 Phase III: Testing and refining programme theories 11
2.3.4 Phase IV: Developing recommendations and disseminating findings 12
3 SETTING 12
4 PARTICIPANTS 12
5 INCIDENTS 13
6 DATA ANALYSIS AND HANDLING 13
6.1 Data Collection – Identifying the literature 13
6.2 Data Handling 15
6.3 Access to Data 16
6.4 Record Keeping 16
7 REGULATORY ISSUES 16
7.1 Peer Review 16
7.2 Ethics Approval 16
7.3 Insurance 16
7.4 Health and Safety 16
7.5 Conflicting Interests or Competing Roles 16
7.6 Monitoring, Audit & Inspections 16
7.7 Protocol Compliance and Amendments 16
7.8 Data Protection and Confidentiality 16
8 DISSEMINATION POLICY 16
10 PROJECT TIMELINE 17
11 REFERENCES 19

1 BACKGROUND

1.1 Challenging behaviour and restrictive interventions

Approximately 1.5 million people in the UK have a learning disability (LD) with approximately 70% of this population also on the autism spectrum (Mental Health Foundation, 2019). A learning disability begins in childhood and primarily affects learning and comprehension (and therefore intellect), whilst autism is a neurological disorder (also called Autistic Spectrum Disorder or ASD) that affects learning delays and social awareness (Connelly, 2007; British Psychological Society, 2012). In addition to ASD, individuals within a primary diagnosis of LD could have a number of other co-morbidities such as epilepsy, schizophrenia and delusional disorders, bipolar affective disorder, depressive disorder, anxiety disorders, specific phobias such as agoraphobia, obsessive compulsive disorder, dementia and personality disorders. It is estimated that approximately 40% of adults with LD also experience mental health problems; this is more than double the rate of mental health problems in the general population (Alexander et al., 2011; Mencap, 2019; McManus et al., 2009). Moreover, adults with LD and/or ASD can present with behavioural challenges that become a risk to themselves or others (Challenging Behaviour Foundation, 2018; Department of Health, 2014a; British Psychological Society, 2012; Royal College of Psychiatrists, 2018). These can include aggressive behaviour directed towards other people, self-harm, and some behaviours which are considered socially unacceptable or offending behaviours. This overlap between learning disabilities, autism, mental illness, and challenging behaviour point to the complex needs of these individuals and the difficulty of addressing these needs within just one service/setting.

The terms 'restrictive practices' and 'restrictive interventions' are often used interchangeably, although they are not always mutually inclusive. Restrictive practices are broader and defined as "making someone do something they don't want to do or stopping someone doing something they want to do" (Department of Health, 2014b: 9). Therefore, these can include, but are not limited to restrictive interventions. There is no one agreed definition of restrictive practices or interventions, but they all advocate for approaches that address a person's human rights. In this proposal, we will use the term 'restrictive interventions' to focus upon practices including observation, seclusion, physical restraint, mechanical restraint, and rapid tranquilisation and other chemical restraint." (Restraint Reduction Network 2019: 159). Restrictive interventions are defined here as "deliberate acts on the part of other person(s) that restrict an individual's movement, liberty and/or freedom to act independently in order to take immediate control of a dangerous situation where there is a real possibility of harm to the person or others if no action is undertaken" (Department of Health, 2014a: 14).

1.2 Evidence explaining why this research is needed now

There is now an emerging political and public pressure to reduce and prevent the use of restrictive practices globally (Wilson et al., 2018; Søndenaa, Dragsten, and Whittington, 2015). National guidance specifies restraint may be used on rare occasions where all de-escalation techniques have failed to eliminate immediate danger or harm to life however, de-escalation, prevention and person-centred therapeutic approaches tackling the root cause of

challenging behaviour should be the first course of action (Department of Health 2014a; NICE, 2015; Restraint Reduction Network, 2019; Care Quality Commission, 2019). Despite this, recent statistics indicate a significant increase of restraint used on adults with LD in English hospitals (NHS Digital, 2018). As a result, the government is being urged to explore in detail the context and reasons for such dramatic increases (Mencap, 2018).

It is clear from our initial scoping review that there is some evidence of the effectiveness of approaches/programmes in reducing the use of restrictive interventions on people with LD, but this is highly variable and limited. Furthermore, there is no evidence or clear indications why these did or did not work. This realist methodology therefore provides a road map for developing theory to understand how interventions work, namely that "causal outcomes follow from mechanisms acting in contexts" (Pawson and Tilly, 1997: 58). Put more simply, interventions occur in specific contexts. The mechanisms at work are, in turn, dependent on that context. As such, Context-Mechanism-Outcome configurations are central to Pawson and Tilley's explanation of 'scientific realism' evaluation (1997) and Pawson's further developments of a realist review methodology (Pawson, 2002; Pawson et al., 2005; Wong et al., 2012). This will provide a useful framework for developing theory that incorporates but extends current knowledge and research in the field.

This review will fill a gap in the evidence by developing programme theories of why approaches work and do not work in preventing and/or reducing the use of restrictive interventions on adults with LD in a variety of NHS and independent sector inpatient and community or residential settings. Specifically, we are interested in uncovering how context influences programme specific theories to guide policy, practice and future research in this area. Interventions reducing challenging behaviour, rather than restrictive interventions tend to dominate the research in this area (McGill et al, 2018; Hayvaert et al., 2014; McQuire et al., 2015; Niven et al., 2018; Hewitt et al., 2016). The assumption that a reduction in challenging behaviour is directly linked to a reduction in restrictive interventions is yet to be evidenced.

We will add to the evidence by understanding the complexity of implementing the range of behavioural support approaches to reduce restrictive practices within complex healthcare settings. The realist review will be informed by a systematic review of effectiveness that the team is currently conducting.

1 RESEARCH QUESTION(S) AND OBJECTIVES

Pawson (2002) sketches out a method for a 'realist review'. A realist review utilises a 'generative' approach to causation whereby it is not 'programmes' that work but instead the underlying reasons or resources that they offer subjects that generate change.

The overall aim of this review is to gain a deeper understanding of what is currently known about how, why, for whom, and in what circumstances approaches to prevent and reduce the use of restrictive interventions on adults with learning disabilities are most successful.

Objectives:

- To use a theory-driven approach to identify the main factors which influence the success or failure of approaches implemented in NHS and independent sector settings with the view to prevent and/or reduce the use of restrictive interventions on people with LD;
- To develop a set of refined programme theories of causal mechanisms and contextual factors linked to short-term, medium-term and long-term outcomes
- To produce recommendations to inform future research, policy and practice and disseminate key mechanisms
- To use extensive consultation with stakeholders to inform theory development, theory testing and recommendations

2 RESEARCH DESIGN

2.1 Review approach

To understand how and why programmes to prevent and/or reduce restrictive interventions used on people with LD in NHS and independent sector settings work, we will undertake a realist review and synthesis of literature (Pawson et al., 2005). Health interventions do not operate in silo, but within complex systems, interacting with personal, interpersonal and environmental variables outside of the programme (Pawson et al., 2005; Connelly, 2007) and, by using a realist approach, one can explore how these interactions impact on the success of a programme. This is realised by identifying and explaining relationships between context (C), mechanism (M) and outcome (O). The basic principle of the realist review method involves starting with a 'rough initial theory', then conducting a systematic but targeted review of literature to 'test' this rough theory, and, finally, re-articulate this in light of the current evidence base (note, this might happen over several iterations until the review reaches 'theory saturation' (Pawson and Tilley, 1997). In realist reviews, theories are articulated in the form of Context-Mechanism-Outcome configurations (CMOs). Identifying the CMOs within a programme enables a better understanding of how, why, for whom, and in what context the programme realises its intended and unintended outcomes in the short, medium and long term.

The realist review is an evidence building, theory-driven and interpretative approach (Pawson, 2006). The basic principle of the realist review method involves starting with a 'rough initial theory', then conducting a systematic but targeted review of literature to 'test' this rough theory, and, finally, re-articulate the 'rough initial theory' in light of the current evidence base. Following the realist methodology, the 'rough initial theory' will take the form of context-mechanism-outcome configurations and begin the identification of middle-range theories underlying the reduction of the use of restrictive interventions.

We have chosen this approach as it is appropriate in dealing with complexity and heterogeneity in study setting, context and design (Pawson et al., 2005), which are characteristics present in the literature we will be reviewing. Approaches implemented to deal with and responses to challenging behaviour, i.e. restrictive practices, are usually complex and commonly delivered in an inconsistent way due to differences in socio-ecological variables that cannot be fully controlled (Williams and Grossett, 2011). We believe that understanding context is key in explaining why current prevention programmes are not consistent in reducing the use of restrictive interventions in LD settings. Another advantage of the realist review approach is that it supports various forms of evidence, with equal importance given to quantitative, qualitative and mixed methods studies (Wong et al., 2012), including grey literature that is usually excluded from more traditional reviews. This gives us the possibility to evaluate underlying theories rather than focusing solely on specific outcome measures.

This realist synthesis will be conducted according to RAMESES guidelines (Wong et al., 2013), and will include four main sequential but iterative phases over 22 months:

- (1) formulating mid-level theories in the form of CMO configurations;
- (2) conducting a systematic search of literature;
- (3) testing and refining programme theories; and
- (4) developing recommendations and disseminating findings.

2.2 Theoretical framework

The review will develop and test a mid-level theory, which will be an explanatory account of how approaches work through the application of (but not exclusive to) theories underpinning behavioural interventions. We recognise, however, the complexity of the interplay between LD, ASD and mental health problems in this population and that our attention needs to span across diagnoses and settings to develop an appropriate theoretical framework. Including a significant stakeholder and expert by experience consultation component within the review will help us carefully consider these complexities and identify or refine candidate theories.

2.3 Review strategy

Our review will be using the accepted four phases of realist synthesis (Pawson, 2006).

2.3.1 Phase I: Formulating mid-level theories in the form of CMO configurations

This phase will involve developing the 'rough initial theory(ies)' that will underpin the rest of the realist review.

Developing CMOs will involve:

- Extensive consultation with a range of experts and stakeholders

- A scoping review of the underpinning academic literature (e.g. policy documents, commentaries, letters, reviews and editorials) that helps us explain the impact and responses to challenging behaviour, including theories underlying behavioural interventions.

Due to the current Covid-19 situation, consultation with stakeholders will now take a more workshop based (online) approach with stages of consultation, CMO development, and reviewing current theories. Stakeholders will include academics, practitioners, service users and carers, commissioners and service providers, members of advocacy groups and charities, and other experts in areas relevant to developing the emerging programme theories.

Step 1: Initial Theory of Change (October 2020):

Dr Szifris will lead a Theory of Change (ToC) workshop with a small number of stakeholders and the wider research team to develop an initial understanding of how specific outcomes are achieved (***Stakeholder workshop #1***). This will include a mapping of a range of possible CMOs and/or mechanisms that relate to the desired outcomes.

Step 2: Developing CMOs and engaging with literature (October-November 2020)

Outcomes of the ToC workshop will be developed into a framework of CMOs that that will provide a framework to map the theoretical and conceptual landscape of restrictive interventions used on people with LD as a response to challenging behaviour. These will then analysed in reference to the wider literature, policy documentation and other commentaries.

Step 3: Wider stakeholder engagement (November-January 2020)

Having developed clear CMOs, the research team will engage with the wider stakeholder group to discuss and refine the initial theory. With the current COVID related situation, we anticipate engaging with the wider stakeholder group online during this period and therefore suggest a staged workshop approach with small numbers of participants (5-7) as opposed to a single, wider workshop including 15-20 individuals. Short (1-2 hour) workshops will be held with each of the following groups separately (***Stakeholder workshops #2***)

- Experts by experience (people with lived experience of LD, ASD)
- Experts by experience (carers/family of people with lived experience of LD, ASD)
- Professionals working in the field, commissioners and policy makers (members of the Advisory Panel)
- Academic experts (research team and their connections)

To be noted that, with regards to experts by experience groups (EEGs) involving people with lived experience, we have established connections with three distinct groups: one for people within forensic settings (led by Thompson); one for people in community settings (led by

Ridley); and one from Learning Disability England (LDE) (led by Bourlet). With the current COVID situation in mind, we are mindful of the implications and appropriateness of working with these sub-groups online.

Completing Step 3 will result in articulating a small number of CMOs (3-5) that reflect genuine mid-level theory (i.e. neither too specific nor too abstract but which reflects the broader behaviours and interventions in which the research is interested).

Step 4: Finalising CMOs (January 2021)

The research team will finalise the CMOs for testing following the consultation period. A final meeting with the research team, advisory panel, and experts by experience will be used to agree the final working of the initial CMOs for testing (***Stakeholders workshop #3***).

Expected outputs from Phase 1: (1) identification of programme theories expressed as CMO configurations to be tested and refined in Phase 3; and (2) submission of the realist review protocol for open access publication

2.3.2 Phase II: Conducting a systematic search of literature

Having developed a rough initial theory in the form of CMOs, the next stage of a realist review involves systematically searching for and sifting through evidence that would assist us in understanding the accuracy of our rough CMOs. In line with the iterative nature of realist synthesis methods (Pawson et al., 2004, 2005), we will finalise the search strategy upon consultation with stakeholders (e.g. members of the advisory panel, experts by experience groups) (***Stakeholders workshop #4***) and the wider research team. This will involve refining study selection criteria, search terms, search syntax and choices of databases. The team acknowledges that adherence to the protocol may change during the systematic review process due to the complexity of managing literature of varying methodologies and the nature of the evidence. Any deviations from the planned protocol will be recorded in the final report.

This phase will involve the following five steps:

Step 1: Study selection criteria

The PICO (Population, Intervention, Comparison, Outcome) framework (Schardt et al., 2007) will be used to inform the search strategy and study selection criteria.

<u>Population</u>: adult (>18 years old) males and females with a primary diagnosis of learning disabilities, who may also have a secondary diagnosis of autism or mental health problems; staff, services or carers that provide support to this specific population.

- A Learning Disability (LD) is a "significant reduced ability to understand new or complex information, to learn new skills (impaired intelligence), with a reduced ability to cope independently (impaired social functioning), which started before

adulthood" (Care Quality Commission, 2019). Mild LDs score an IQ of 50-70, moderate 35-50, severe 20-35 and profound less than 20 (World Health Organisation, 2019).

- Adults with LD and on the autistic spectrum (autism/autistic spectrum disorder) will also be included. Autism or Autistic Spectrum Disorder (ASD) is a complex neural disorder affecting development, usually diagnosed in childhood, with persistent impairment in social interaction, relationships, arousal impairments, verbal and non-verbal communication, and restricted/repetitive behaviours (American Psychiatric Association, 2013).
- Adults with LD and a mental health diagnosis will also be included. The search will include (but not exclusive to) mental health problems such as schizophrenia, bipolar disorder, depression, anxiety disorders, specific phobias, agoraphobia, obsessive compulsive disorder and dementia – problems which have been identified as common in adults with LD.

<u>Intervention</u>: Any programmes or discreet interventions designed to prevent or reduce the use of restrictive interventions. Examples of multi-component/complex programmes or approaches include: the Safewards model (Bowers et al., 2014) and the RAID and Positive Behaviour Support (PBS) approaches developed in the UK (Gore et al., 2013), the Engagement model (Borckardt et al., 2007) and the Six Core Strategies (Huckshorn, 2005) and the 'No Force First' (Ashcraft and Anthony, 2008; Ashcraft, Bloss, and Anthony, 2012) developed in the US. Examples of 'simple'/discreet interventions include: staff training and de-escalation, debriefing, staff education and e-learning; review (post incident), sensory modulation (environmental), risk assessment (MAS-M, Broset, DASA), data analysis and text mining; joint crisis plans, open doors policy, behavioural/non-pharmacological interventions, Methodical Work Approach (MWA), clozapine, Relational Neurobehavioral Approach (RNA), recovery-based practice, rapid restraint analysis (RRA), post-seclusion counselling.

<u>Comparison</u>: Other interventions or treatment as usual.

<u>Outcomes</u>: The national and international consensus – whether in policy, guidelines or the literature – is that there is an urgent need to minimise the use of restrictive interventions on vulnerable people with LD. A reduction in restrictive interventions will therefore be a candidate primary outcome for this review. We will include all types of restrictive interventions, duration and frequency. Examples include: all forms of restraint, seclusion, observation, segregation, rapid tranquilization and other types of coercive medication (IM, PRN), restriction by default, clinical holding, blanket rules/restrictions. However, caution will be used when selecting outcomes to avoid outcome reporting bias. Authors of included papers will be contacted if it is unclear whether significant and non-significant restrictive interventions minimisation outcomes were recorded.

The review process will encompass an iterative identification of outcomes that are important to stakeholders, in particular service users and carers. Other candidate outcomes will include: service users' safety (including incidents of aggression; self-harm; injury); service user and carer satisfaction with the service; staff attitudes; staff safety, burnout, stress.

The full inclusion and exclusion criteria will be fully defined following the initial scoping review and the stakeholder consultation.

We will include all published and grey evidence of any study design including single case studies, RCTs, quasi-experiments and observational studies looking at the impact of any approaches implemented to address challenging behaviour and restrictive interventions. We anticipate mixed-method studies and qualitative studies will provide evidence of facilitators and barriers to the implementation and success of such approaches. This will include participants' views and experiences of preventative or reduction programmes.

For the other steps within this phase, please refer to Section 6.1.

<u>Expected outputs from Phase 2:</u> (1) eligible evidence to test and refine the initial CMOs; and (2) developed data extraction forms for different types of evidence and content of CMOs.

2.3.3 Phase III: Testing and refining programme theories

The analytical task is in aligning the extracted evidence in relation according to the relationships between Mechanisms (e.g. underlying processes and structures), Contexts (e.g. diagnoses, setting, organisational configurations), and Outcomes (i.e. intended and unintended consequences and impact) with the initial programme theories. Rycroft-Malone et. al. (2012) have developed an approach to synthesis which builds on Pawson's work (2006) and the principles of realist enquiry, including the following steps:

1. organisation of extracted information into evidence tables representing the different bodies of literature;

2. theming by individual reviewers across the evidence tables to identify demi-regularities (reoccurring patterns);

3. comparison of reviewers' themes and linking agreed demi-regularities seeking confirming and disconfirming evidence;

4. refinement or development of new explanatory middle-range theories expressed as CMO configurations

Realist synthesis is a time consuming and resource intensive phase within the project, as it will involve individual reflection, team discussion and further consultation with key stakeholders, e.g. practitioners, policy makers, charity workers and experts by experience (people with lived experience and carers). The view of stakeholders will be sought via a follow

up workshops (***Stakeholders workshop #6***). This will include in-depth discussion of the findings to develop and confirm the resultant hypotheses. These will act as synthesised statements of findings around which a narrative can be developed summarising the nature of the context, mechanism and outcome links, and the characteristics of the evidence underpinning them. We are hoping to be able to organise face to face workshops where we present our findings and we validate them by consulting with key experts, but smaller online workshops will be considered as an alternative if the situation regarding social isolation/COVID does not improve by that time. A refined set of hypotheses with accompanying evidence-based narrative will be produced.

We will also re-visit eligible evidence, extracted data and perform additional searches, if there are any unanticipated development of new explanatory middle-range theories during the synthesis stage.

Expected outputs from Phase III: (1) an evidence-based framework indicating what works for whom and in what context in relation to reducing the use of restrictive interventions on people with learning disabilities and autism or mental health co-morbidities.

2.3.4 Phase IV: Developing recommendations and disseminating findings

A final stakeholder consultation will take place to help produce recommendations for policy and practice (***Stakeholder workshop #7***). There is a possibility of limited evidence for any proposed theory, thus recommendations could also highlight the strength of evidence in this area. This project might highlight theories, interventions or mechanisms that need to be tested with further, more robust research, e.g. randomised trials (cluster, stepped wedge).

We will finalise the project by holding a ***knowledge mobilisation event*** open to a wide range of key stakeholders, academics, practitioners, people with lived experience, policy makers, commissioners, to present the results and emerging recommendations and identify possible barriers to the adoption and implementation of evidence-based findings.

Expected outputs from Phase IV: (1) refined/actionable recommendations; (2) realist review final report; and (3) paper to be submitted in open access peer reviewed journal.

3 SETTING

This study is fully taken place at MMU premises. It comprises of the already published literature.

4 PARTICIPANTS

Human participants or otherwise will not be part of this project. This project is a realist review based entirely on secondary research. We will, however, engage with key stakeholders (e.g. professionals and people with lived experience) who will guide the scope of the review by providing their input on refining specific parts of the research that includes the full eligibility criteria for the selection of evidence, data extraction forms and the refinement of programme theories.

5 INCIDENTS

There will be no research participants involved in this project.

6 DATA ANALYSIS AND HANDLING

As this is a review, we will only be examining paper-based materials.

6.1 Data Collection – Identifying the literature

The search strategy will be developed with the help of a clinical librarian, with input from the project team and stakeholders to identify relevant published and unpublished evidence. We will also consult search strategies from previous relevant reviews. A comprehensive overall search be will used to search the following databases from 2000-onwards to ensure relatively recent, but sufficient evidence: Cumulative Index to Nursing and Allied Health Literature (CINAHL), EMBASE, Education Resources Information Centre, MEDLINE, PsycINFO, PubMed and Web of Science.

<u>Searching for grey and unpublished literature</u>

Supplementary searches will be used to search for grey and unpublished literature. We will conduct a broad search on Open Grey (http://www.opengrey.eu/), Grey Literature Report (http://www.greylit.org), World Health Organisation (http://www.who.int/en/) and Google. We will attempt to identify any evidence from as many sources as possible. That includes asking EEG and AP members to identify any known literature and useful websites and organisations to contact. The results from these methods of searching will be saved to record the source data (URL, organisation or expert), the date found/contacted and the references identified via that method.

In addition to searching reference lists of eligible evidence identified through electronic database searching, a purposive and iterative approach will be employed following the CLUSTER (Citations, Lead Authors, Unpublished materials, Scholar searches, Theories, Early Examples and Related Projects) methodology (Booth et al., 2013) as illustrated in Table 1. This approach will involve identifying the 'key pearl citations' (i.e. key evidence in the topic area) to identify additional relevant outputs that may include 'sibling' studies (i.e. evidence from the same study – for example, qualitative studies associated with an RCT) or 'kinship' studies (i.e. theoretically associated) that inform contextual elements. This method overcomes one of the limitations of database searching by aiming to identify additional evidence linked with a study of interest, instead of potential evidence simply using the same terminology.

Table 1. The CLUSTER approach

Element	Search procedure	Sources
**C**itations	Identify at least one 'key pearl' through consensus with review team	Preliminary searches of databases and grey literature
**L**ead authors	Check reference list of 'key pearl', conduct lead author search	Full text of 'key pearl', search of reference management collection, Google (e.g. institutional repository, author publication webpage)
**U**npublished materials	Make contact with lead author	Email
**S**cholar searches	Citation searches on 'key pearl' and other relevant studies. Conduct search of 'project name'	Web of Science/Google Scholar
**T**heories	Follow up 'key pearl' and other cluster documents for citations of theory. Recheck for mention of theory in titles/abstracts/keywords, iterative searches for theory in combination with condition of interest	Full text of 'key pearl', search of reference management collection, databases
**E**arly examples	Follow up key pearl citation and other cluster documents for citations to project antecedents and related projects	Full text of 'key pearl'
**R**elated projects	Conduct named project and citation searches for relevant projects identified from cluster documents, seek cross case comparisons by combining project name/identifier for cluster with project name/identifiers for other relevant projects	Web of Science/Google Scholar, databases

Step 3: Screening process

The results of the database searching and selection process will be reported using the Preferred Reporting Items for Systematic Reviews and Meta-Analyses (PRISMA; Moher et al., 2009). Two reviewers will independently screen all titles and abstracts for potentially identify relevant evidence. Then full-text versions of potentially relevant evidence will be assessed for inclusion using the eligibility criteria. Disagreements will be resolved by discussion with a third reviewer where necessary. The reasons for exclusions at full-text level will be recorded following the PRISMA flowchart.

Step 4: Quality assessment

The methodological quality of included evidence will be assessed using appropriate tools based on study design. For example, Critical Appraisal Skills Programme (CASP, 2018) will be used to assess qualitative studies, mixed-methods studies will be assessed using the Mixed Methods Appraisal Tool (MMAT) and grey literature will be assessed using the Authority, Accuracy, Coverage, Objectivity, Date, Significance (AACODS) checklist (Tyndall, 2010). The appraisal of evidence will be assessed by one reviewer and independently verified by a second reviewer. Any disagreements will be resolved through consensus and if necessary, a third reviewer will be consulted for arbitration. Studies will not be excluded by their methodological quality as evidence in realist reviews is based on what knowledge they bring, we are however, conducting quality assessment to ensure transparency (Kastner et al., 2011).

Step 5: Data extraction

Selected studies meeting the full criteria will be read twice before data extraction onto bespoke data extraction forms (developed based on the content of the programme theories that emerge from Phase 1) in order to collate the evidence on CMOs. The stakeholders and wider research team will inform the data extraction strategy as it is anticipated data extraction will differ based on the type of evidence, content of CMOs and the underlying review question. We will conduct a workshop with key members of the RMG, AP and EEGs to agree on components to be extracted for the realist review (***Stakeholders workshop #5***).

In conjunction with the data extraction forms, we will extract study aims, methods used and characteristics of the population from all eligible evidence. This will include but not be limited to: publication characteristics (e.g. country of data), study design (e.g. review, observational, cohort, qualitative studies), type of interventions, context of intervention, mechanisms generated, descriptions of all outcomes and author interpretations (e.g. any theories or mechanisms postulated by study authors) that explain the outcomes of interventions. Data will be extracted independently by one reviewer and checked for accuracy by second reviewer. Any disagreements will be resolved through consensus and a third reviewer will consult where necessary. Data from multiple related publications of single studies will be extracted and reported as a single reference. We will attempt to contact corresponding authors for missing data, where possible.

The extracted data will be tabulated to summarise studies and allow evidence to be meticulously mapped against the initial programme theories. This will also allow the identification of any new programme theories.

6.2 Data Handling

Data that is already available in the public domain (i.e. secondary research) will be managed in Covidence, a reference manager.

6.3 Access to Data

Besides the research team, access will be granted to authorised representatives from the University to permit study-related monitoring, audits and inspections. The authority representatives at NIHR will reserve the right to have access to and use the research data compiled during the course of the research.

6.4 Record Keeping

No record keeping will be carried out as we are only seeking evidence that is already published or publicly available.

7 REGULATORY ISSUES

7.1 Peer Review

Internal formal peer review of the project was carried out by members in the Faculty of Health, Psychological and Social Care at MMU. Given that this project is a result of competitive NIHR funding, it has been formally reviewed by a panel of independent reviewers.

7.2 Ethics Approval

Ethics and governance approvals will be sought from Manchester Metropolitan University. We will use the Ethics Online System to submit the ethics application and any necessary amendments.

7.3 Insurance

Not applicable

7.4 Health and Safety

Health and safety concerns will be very minimal as there are no research participants involved in this research.

7.5 Conflicting Interests or Competing Roles

There are no declared conflicting interests or competing roles.

7.6 Monitoring, Audit & Inspections

This is a review that is summarising evidence that is already in the public domain.

7.7 Protocol Compliance and Amendments

We understand that any deviation from the approved protocol will require amendments. We will do this via the Ethics Online System by creating a sub-form.

7.8 Data Protection and Confidentiality

There is no primary research data being collected in this project.

8 DISSEMINATION POLICY

The data generated will be owned by MMU. Members of the project research team will be the authors on any of published outputs such as the NIHR report and other peer reviewed publications. The authors will acknowledge the funding body (i.e. NIHR) in all publications

and will send one draft copy of the report/proposed publication to the NIHR at the same time as submission for publication or at least 28 days before the date intended for released, whichever is earlier. The views expressed in any publications will be those of the author(s) and not necessarily those of the NHS, NIHR or the Department of Health and Social Care.

The final report will be published on the NIHR webpage in the *Health Services and Delivery Research (HS&DR)* Journal (https://www.journalslibrary.nihr.ac.uk/hsdr/#/). All reports published in HS&DR are open access. Results will also be published in other peer-reviewed journals, e.g. Journal of Intellectual Disabilities and Offending Behaviour, Journal of Adult Protection, Journal of Applied Research in Intellectual Disabilities.

Researchers, experts by experience, support groups and healthcare professionals, commissioners and the wider public will be invited to attend project conference to find out and engage with the results from the study. The event will be widely advertised through the NHS internal communications, care homes and private hospitals, practitioner journals and social media. Key note presentations will be video captured and disseminated through social media (You Tube, Vimeo and blogs) to maximise access for those who cannot attend and for impact in the medium/long term and for reaching out to wider audiences. In addition, video or audio podcasts of the project findings will be similarly produced and disseminated to maximise public access.

Service providers and commissioners will be provided with the project outputs (listed above) to disseminate through their internal communications (e.g. bulletins, e-mail updates, intranet). A lay summary of findings (easy read accessible in different formats) will be developed with the aid of people with LD to make findings accessible to service users and their families. This will be distributed to people with lived experience using the project developed networks.

The findings will also be designed into an interactive poster with a QR code directly linking audiences to the YouTube and Vimeo videos where they can stream research findings and the policy and practice implications live to computers and hand-held devices. The poster will be taken to local, regional and national conferences and disseminated trough all social media platforms. In February 2018, BBC One Breakfast reported on the alarming use of restraint practices in LD settings. On completion of the project, we will approach the BBC to do a follow up interview based on our research findings.

10 PROJECT TIMELINE

	LD REVIEW GANTT CHART (Sept 2020 – Jun 2022)	MONTH																								
		PREP				1	2	3	4	5	6	7	8	9	1	1	1	1	1	1	1	1	1	1	2	2	2
Prep	**Setting up**																										
	Complete MIS Tasks																										
	• Confirm & Update Website Information, PROSPERO																										
	• Submit Collaboration/Other Agreement																										
	• Upload Project Management Plan																										
	• Submit Protocol to NIHR																										
	• Provide Evidence of Ethical Approval																										
	RA Recruitment, Protocol confirmation, Ethics Application																										
	Project launch																										
	Submit Protocol for open access publication																										
Phase 1	**Formulate mid-level theories in the form of CMO configurations**																										
	Initial search strategy																										
	Initial/scoping review																										
	Theory of Change/ToC (Stakeholder workshop #1) (RMG+AP+EEG																										
	Develop CMOs																										
	Confirm CMOs (Stakeholder workshop #2) (RMG+AP+EEG																										
	Final CMOs (Stakeholder workshop #3) (RMG+AP+EEG meeting#3)																										
Phase 2	**Conduct a systematic review of literature (Feb – Nov'21)**																										
	Searches, screening, data extraction (incl. Stakeholder workshops																										
	Synthesis [CMO] - tables																										
	Develop/confirm candidate programme theory																										
Ph as	**Test and refine programme theories (Dec'21 - Jan'22)**																										
	Refined theory (incl. Stakeholder workshop #6) (RMG+AP+EEG																										
Phase 4	**Develop recommendations and disseminate findings (Feb –**																										
	Recommendations (Stakeholder workshop #7) (RMG+AP+EEG																										
	Dissemination Event																										
	NIHR report + paper submitted OA journal (Jun'22)																										
	Other dissemination (podcasts/conferences/media/events)																										

11 REFERENCES

Mental Health Foundation (2019) Learning disability statistics: autism | Mental Health Foundation. [Online] [Accessed 29th March 2019] https://www.mentalhealth.org.uk/learning-disabilities/help-information/statistics/learning-disability-statistics-/187690

Connelly, J., B. (2007) 'Evaluating Complex Public Health Interventions: Theory, Methods and Scope of Realist Enquiry.' J Eval Clin Pract. 13, 935-941.

British Psychological Society (2012) Autism the Nice Guideline on Recognition, Referral, Diagnosis And Management of Adults on The Autism Spectrum. [online] [Accessed 25th March 2019] https://www.ncbi.nlm.nih.gov/pubmed/26065067

Alexander, R. T., Hiremath, A., Chester, V., Green, F. N., Gunaratna, I. J., & Hoare, S. (2011) Evaluation Of Treatment Outcomes from A Medium Secure Unit for People with Intellectual Disability. Advances in Mental Health and Intellectual Disabilities. 5 (1) 22-32.

Mencap (2019) Learning Disability - Mental Health Research- Mencap. [Online] [Accessed 29th March 2019] https://www.mencap.org.uk/learning-disability-explained/research-and-statistics/health/mental-health

McManus, S, Meltzer, H, Brugha, T, Bebbington, P. and Jenkins, R., (2009) Adult Psychiatric Morbidity in England, 2007: Results of A Household Survey. The Health and Social Care Information Centre, Social Care Statistics.

Challenging Behavior Foundation (2018). What is Challenging Behaviour. Drafts. The Challenging Behaviour Foundation, UK. [Online] [Accessed 25th March] Available at: https://www.challengingbehaviour.org.uk/about-us/about-challenging-behaviour/what-is-challengingbehaviour.html

Department of Health (2014a) Positive and Proactive Care: Reducing the Need for Restrictive Interventions Prepared by The Department of Health. [online] [Accessed 25th March, 2019] https://assets.publishing.service.gov.uk/government/uploads/system/uploads/attachment_data/file/300293/JRA_DoH_Guidance_on_RP_web_accessible.pdf

Royal College of Psychiatrists (2018). Restrictive Interventions in In-Patient Intellectual Disability Service. How to Record, Monitor and Regulate. [online] [Accessed 25th March 2019] https://www.rcpsych.ac.uk/docs/defaultsource/improving-care/better-mh-policy/college-reports/college-report-cr220.pdf?sfvrsn=c74ad0e4_2

Department of Health (2014b) A Positive and Proactive Workforce: Skills for Care and Skills for Health. [online] [Accessed 29th March 2019] https://www.skillsforcare.org.uk/Documents/Topics/Restrictive-practices/A-positive-andproactive-workforce.pdf

Restraint Reduction Network (2019) Ethical Training Standards to Protect Human Rights and Minimise Restrictive Practices. [online] [Accessed 29th March 2019] https://www.researchgate.net/publication/329686659_Ethical_training_standards_to_protect_human_rights_and_minimise_restrictive_practices_D_R_A_F_T_Restraint_Reduction_Network_RRN_Training_Standards_2018_D_R_A_F_T_Restraint_Reduction_Network_RRN_Traini

Wilson, C., Rouse, L., Rae, S. & Kar Ray, M., (2018) ‘Mental Health Inpatients’ and Staff Members’ Suggestions For Reducing Physical Restraint: A Qualitative Study’. Journal of Psychiatric and Mental Health Nursing. 25 (3), 188-200.

Søndenaa, E., Dragsten, F. & Whittington, R. (2015) ‘Practitioner Explanations for The Increasing Use of Restraint Measures in The Care of People with Intellectual Disabilities in Norway 2000-11’. Journal of Policy and Practice in Intellectual Disabilities. 12 (1), 58-63

NICE (2015) Challenging Behaviour and Learning Disabilities: Prevention and Interventions for People with Learning Disabilities who Behaviour Challenges. [online] [Accessed 10th April 2019] https://www.nice.org.uk/guidance/ng11

Care Quality Commission (2019) Our Review of Restraint, Seclusion and Segregation. [Online] [Accessed 25th March, 2019] https://www.cqc.org.uk/publications/themes-care/our-review-restraint-seclusion-segregation

NHS Digital (2018) Restraints, assaults and self-harm data 2016-2018 - NHS Digital. [Online] [Accessed 29th March 2019] https://digital.nhs.uk/data-and-information/find-data-and-publications/supplementary-information/2018-supplementary-information-files/lda---restraints-assaults-and-self-harm-data-2016-2018

Mencap (2018) Mencap responds to BBC File on 4 Programmes on Transforming Care | Mencap. [Online] [Accessed 29th March 2019] https://www.mencap.org.uk/press-release/mencap-responds-bbc-file-4-programme-transforming-care

Pawson, R., Tilly, N. (1997) Realist Evaluation. Sage: London.

Pawson, R. (2002) Evidence-Based Policy: The Promise Of ‘Realist Synthesis’. Evaluation. 8 (3), 340–358.

Pawson, R., Greenhalgh, T., Harvey, G., & Walshe, K. (2005). ‘Realist Review – A New Method of Systematic Review Designed for Complex Policy Interventions’. Journal of Health Services Research and Policy. 10, 21–34.

Wong, G., Greenhalugh, T., Westhorp, G., Pawson, R. (2012) ‘Realist Methods in Medical Education Research: What They Really are and What They can Contribute?’ Medical Education. 46 (1), 89-96.

McGill, P., Vanono, L., Clover, W., et., al. (2018) 'Reducing Challenging Behaviour of Adults with Intellectual Disabilities in Supported Accommodation: A Cluster Randomized Control Trial of Setting Wide Positive Behaviour Support'. Research in Developmental Disabilities. 81 (2018), 143-154.

Hayvaert, M., Maes B., Onghena, P. (2014) 'A Meta-Analysis of Intervention Effects on Challenging Behaviour Amoung Persons with Intellectual Disabilities'. Journal of Intellectual Disability Research. 54 (7), 634-649.

McQuire, C., Hassiotis, A., Harrison, B., Pilling, S. (2015) 'Pharmacological Interventions for Challenging Behaviour in Children with Intellectual Disabilities: A Systematic Review and Meta-Analysis'. BMC Psychiatry. 26 (15), 303.

Niven, A., Goodey, R., Webb, A. & Shankar, R. (2018) 'The Use of Psychotropic Medication for People With Intellectual Disabilities and Behaviours That Challenge in The Context of a Community Multidisciplinary Team Approach'. British Journal of Learning Disabilities.

Hewitt, O., Keeling, N. & Pearce, M. (2016) Training A Family in Physical Interventions as Part of A Positive Behaviour Support Intervention for Challenging Behaviour. British Journal of Learning Disabilities.

Pawson, R. (2006) Evidence-Based Policy: A Realist Perspective. London: Sage.

Williams, D. E. & Grossett, D. L. (2011) 'Reduction of Restraint of People with Intellectual Disabilities: An Organizational Behavior Management (OBM) Approach'. NCBI Pub Med. 32 (6), 2336-2339.

Wong, G., Greenhalugh, T., Westhorp, G., Cuckingham, J., Pawson, R. (2013) RAMESES Publication Standards: Realist Synthesis. BCM Medicine. 11 (21). [online] [Accessed 9th April, 2019] https://bmcmedicine.biomedcentral.com/track/pdf/10.1186/1741-7015-11-21?site=bmcmedicine.biomedcentral.com

Schardt, C., Adams M.B., Owens, T., Keitz, S., Fontelo, P. (2007). Utilization of the PICO framework to improve searching PubMed for clinical questions. BMC Medical Informatics and Decision Making. 7(16)

World Health Organisation. (2019) Definition: Intellectual Disability. [online] [Accessed 29th March 2019] http://www.euro.who.int/en/health-topics/noncommunicable-diseases/mental-health/news/news/2010/15/childrensright-to-family-life/definition-intellectual-disability

American Psychiatric Association. (2013) Diagnostic and statistical manual of mental disorders (5th ed.). Washington, DC: Author.

Bowers, L. et. al., (2014) 'Safewards: The Empirical Basis of The Model and A Critical Appraisal'. Journal of Psychiatric and Mental Health Nursing. 21 (4), 354-364.

Gore, N., McGill., Toogood, S., et al. (2013) Definition and scope for positive behaviour support. International Journal of Positive Behavioural Support, 3 (2). pp. 14-23

Borckardt, J. J., Grubaugh, A. L., Pelic, C. G., Danielson, C. K., Hardesty, S. J., Frueh, B. C. (2007). Enhancing patient safety in psychiatric settings. Journal of Psychiatric Practice, 13, 355-361.

Huckshorn, K.A. (2005) Six core strategies to reduce the use of seclusion and restraint planning tool. National Association of State Mental Health Program Directors.

Ashcraft, L., Anthony, W. (2008) Eliminating seclusion and restraint in recovery-oriented crisis services. Psychiatric Services, 59(10), 1198-1202

Ashcraft, L., Bloss, M., Anthony, W. (2012) The development and implementation of 'No Force First' as a best practice. Psychiatric Services, 63(5), 415-417

Rycroft-Malone, J, Burton C, Hall B, et al. (2012) 'Improving Skills and Care Standards in The Support Workforce For Older People: A Realist Review'. BMJ Open 2014;4:e005356

Booth, A, Harris, J., Croot, E., Springett, J., Campbell, F., Wilkins, E. (2013) Towards a methodology for cluster searching to provide conceptual and contextual 'richness' for systematic reviews of complex interventions: case study (CLUSTER). BMC Medical Research Methodology, 13(118)

Critical Appraisal Skills Programme. (2018) CASP Qualitative Checklist. [online] [Accessed 10th June, 2020] Available at: https://casp-uk.net/casp-tools-checklists/

Tyndall, J. (2010) AACODS Checklist. Flinders University. [online] [Accessed on 10th June, 2020] Available at: https://canberra.libguides.com/c.php?g=599348&p=4148869

Kastner, M., Estey, E., Perrier, L, et al. (2011) Understanding the relationship between the perceived characteristics of clinical practice guidelines and their uptake: protocol for a realist review. Implementation Science, 6(69)
