# Supplementary material for: Approaches used to prevent and reduce the use of restrictive practices on adults with learning disabilities: Protocol for a realist review
Source: PLoS One. 2022 Sep 28;17(9):e0270028. doi: 10.1371/journal.pone.0270028 (PMC9518871; doi:10.1371/journal.pone.0270028)
Supplement: Attachment — Submitted filename: Response to Reviewers [03.11.2021].docx [file pone.0270028.s003.docx]

Response to Reviewers

Letter responding to each point raised by editor and reviewers.

Thank you for identifying both strengths and weaknesses of our protocol and considering it for publication. We have addressed all comments from the reviewer, which were also highlighted by the editor. Our response is included in this document and the amended manuscript reflects the proposed changes.

|  | **Editor** | **Response** |
| --- | --- | --- |
| 1. | Restructure the introduction to better clarify the general approach, and the inclusion and exclusion criteria | We have restructured the introduction to clarify the prevalence of use of restrictive practices particularly for people with learning disabilities and the type and scope of approaches used to reduce such practices for people with learning disabilities, supported by additional evidence (new citations and references). This strengthens the rationale for the review.  See change in text and new citations lines: 60-62 (p. 3), 66, 72-79 (p. 4), 94-130 (pp. 5-6) in the revised manuscript with track changes. Some references were deleted and new ones included. |
| 2. | Additional information on methodological aspects of the study (such as the inclusion of some parts of the “patient and public involvement" section in the Methods | This has now been addressed. The section on inclusion of experts by experience/people with lived experience, i.e. patients and carers has now been moved at the end of the Methodology section (lines 309-322, p. 14 in the revised manuscript with track changes).  A paragraph highlighting the benefits of including the views of experts by experience has been included at the end of the rationale section (lines 134-137, p. 6) |
|  | **Reviewer’s comments** | **Response** |
| 3. | My main concern relates to the targeted population, adults with learning disabilities. In the introduction, there seems to be a blurring and constant back and forth between articles referring to interventions in a psychiatric context and articles referring to interventions in a context of learning disabilities. So I believe that the introduction should be restructured and deepened, which will allow a better understanding of the approach, and the inclusion and exclusion criteria. | Thank you for pointing out this limitation.  We have restructured the introduction to clarify the prevalence of use of restrictive practices particularly for people with learning disabilities and the type and scope of approaches used to reduce such practices for people with learning disabilities, supported by additional evidence (new citations and references). This strengthens the rationale for the review.  See change in text and new citations lines: 60-62 (p. 3), 66, 72-79 (p. 4), 94-130 (pp. 5-6) in the revised manuscript with track changes. Some references were deleted (mental health specific) and new ones included (LD/ID specific). |
| 4. | Title: “approaches” is preferred above programmes and interventions. One concept should be chosen and used throughout the paper. | This terminology (approach/approaches) is now used across the review protocol. |
| 5. | Introduction: The third paragraph (70-82) is weaker and needs to be better integrated. The authors refer to “restrictive practices in mental health or learning disability settings”, but differences and similarities amongst them should be explained. | As specified above, we have now identified the key approaches used in these settings and summarised the evidence to support these. A parallel with evidence re mental health setting has been drawn. |
| 6. | 83-85: “focused on their effectiveness on the reduction of behaviour that challenges, not necessarily the reduction of use of restrictive practices”: I disagree with this, especially as the authors base their arguments on three papers from the same research team. | This paragraph has now been deleted and rephrased in line with the evidence. |
| 7. | 86: The previous paragraph introduces different approaches, but not "multimodal programs" when indeed, a quick review of the literature can identify evidence to this effect. This is an example where I think the authors can restructure the introduction | This paragraph has now been rephrased in line with the evidence. |
| 8. | Some parts of the “patient and public involvement” section should be in the Methods. The need for patient and public involvement would benefit from being linked to the introduction, but the explanation of this kind of involvement in the realist review shoud be presented in the Methods section | This has now been addressed. The section on inclusion of experts by experience/people with lived experience, i.e. patients and carers has now been moved at the end of the Methodology section (lines 309-322, p. 14 in the revised manuscript with track changes).  A paragraph highlighting the benefits of including the views of experts by experience has been included at the end of the rationale section (lines 134-137, p. 6) |
| 9. | The section “Objectives” does not include objectives but the aim of the study, but this one is clear. | The title of the section has now been renamed ‘aim of the review’. If needed, we can include specific objectives, but we feel the main aim should suffice. |
| 10. | Design: The design is well-presented and described.  143-144: What is the link between reference 44 and the sentence? This reference should be in the introduction.  My main concern, as stated before, is on the targeted population: for step 1, is the focus of search goes “beyond specific interventions” as well as populations? It is not clear what will be specific to the targeted population.  For step 2, what is the process for the grey literature search? How will unpublished evidence be obtained?  188-195: Pawson’s method was first published in 2005, and the science of knowledge syntheses has evolved enormously since then. Although according to the realist review approach, the quality assessment with validated tools is not mandatory, several researchers are now proposing to carry out a quality assessment even if it was not previously required in the method. I suggest the authors refer to JBI’s Critical Appraisal Tools.  Some steps need more precision. For example, is the eligibility of evidence assessment based on the full paper, abstract, etc…  Step 3: can the authors be more precise on how they will code something like the “mechanisms”, since it’s “usually hidden”? | The citation was erroneously included. This has now been removed.  Lines 199-203 have now been deleted to prevent confusion.  CLUSTER will be used to attempt to identify grey literature by contacting authors of eligible studies and searching on institutional repositories. Additionally, a free hand search on ProQuest and OpenGrey will be considered if CLUSTER yields insufficient grey literature.  We have stated that quality appraisal of eligible articles will not be carried out as it is anticipated the search will yield opinion pieces, editorials and other forms of evidence that cannot be appraised using traditional quality assessment tools, which includes JBI’s tools. Additionally, it is important to acknowledge that we’re not including data based on the assessment of the methodology in which determined a particular effect size. In realist reviews, it is anticipated we’re only going to be using little bits of information from a variety of sources that cannot be formally assessed. For example, using authors’ interpretations. Evidence in realist reviews are typically judged on two domains: relevance and rigour. We have embellished this particular section in how specifically we’re categorising relevance and rigour (lines 258-268).  Eligibility of articles will be assessed at two stages: title/abstract and then at full-text level (line 275).  To address the comment relating to coding the mechanisms, it is important to acknowledge that this process is interpretative. We have therefore added the following sentences to reiterate this: “The coding will follow a realist, explanatory logic starting from relevant outcomes. Attempts will then be made to interpret and explain how healthcare professionals respond to resources provided to them (the mechanisms) from different approaches aimed at reducing restrictive practices. The specific contexts or circumstances will then be identified when these mechanisms are likely to be triggered. ” (lines 292-299) |
| 11. | I’m not sure if the study’s timeline presented was adjusted. | The timeline of this review has not changed and is still expected to run for a 22-month period from September 2020. The searches, however, were conducted in July instead of May. This change has now been reflected in the main text (line 221) |
